# 

**Published:** 1888-02-18

**Authors:** 


CONTENTS.

**The Market Value of Conscience** - - - - - 343

**Words of Consolation.** Chapter LXVI.—The Sufferings of Christ - - - - - - - - 344

**The Doctor's Pulpit.**—Brains at a Premium - - - 345

**Convalescent Homes in the Eastern Counties.** By Our Roving Correspondent - - - - - 346

**Aids to Nursing.** By M. M Basil, M.A. M.B., Author of "The Commoner Accidents to Life and Limb" - - 347

**The Students' Column.**—University of London: Preliminary Scientific (M.B.) Examination, etc. - - - - 348

**Love-Lights.** By J. A. Thomson, M.A., Lecturer on Zoology in the University of Edinburgh - - - - - 349

**The National Pension Fund for Nurses** - - - 349

**Notes and News.**—City of London Lying-in Hospital.—Proposed Cottage Hospital, Falkirk.—The Late Dr. Woollett and the Newport Infirmary.—British Nurses' Association Meeting.—A Canard Contradicted - - - - - - - 350

**Everybody's Page** - - - - - - - - 352

**Annotations.**—A New Occupation for Women.—The Morals of the Charitable.—What Dreams are made of - - - 353

**Rachel Challice's Vow.** By Lina Nevill, Author of "A Future on Trust," etc. - - - - - - 354

**The Hospitals Association:** The Relation of the Medical School to the Hospital. By Timothy Holmes, F.R.G.S. - 356

**Amusements with Profit for all Ages.**—For Men and Women.—Children's Corner.—Puzzles, etc. - - - 358

The average issue of "The Hospital" now exceeds **10,000 COPIES WEEKLY,** and the circulation is steadily increasing

**CITY OF LONDON LYING-IN HOSPITAL,**

